# Effects of Moderately Reduced Dietary Net Energy on Growth Performance, Meat Quality and Intestinal Barrier Function in Growing Pigs

**DOI:** 10.3390/vetsci13060515

**Published:** 2026-05-26

**Authors:** Dexin Zhao, Haoliang Chai, Taibiao Wang, Shaoshuai Zhang, Weiqi Peng, Chengjun Hu, Renlong Lv

**Affiliations:** 1 Tropical Crop Genetic Resource Research Institute, Chinese Academy of Tropical Agricultural Sciences, Haikou 571101, China; 2 College of Animal Science and Technology, Northeast Agricultural University, Harbin 150030, China; 3 College of Animal Science and Technology, Henan University of Science and Technology, Luoyang 471023, China; 4 Gembloux Agro-Bio Tech, University of Liège, B-5030 Gembloux, Belgium

**Keywords:** growth performance, intestine health, meat quality, net energy, pigs

## Abstract

This study assessed the impacts of dietary net energy (NE) levels on growth performance, meat quality and intestinal health in Tunchang Black pigs. Forty-eight pigs with an initial body weight of 11 kg were allocated into four groups and fed diets containing 10.65 (N1), 10.15 (N2, control), 9.65 (N3) and 9.15 (N4) MJ/kg NE respectively over a 42-day experimental period. The N4 group presented lower average daily gain and carcass weight relative to the N1 group, while no significant differences in growth performance were detected between N2 and N3. The N3 group displayed superior meat quality characteristics, manifested as decreased contents of saturated fatty acids (C10:0, C12:0), elevated selenium concentration, upregulated expression of slow-twitch myosin heavy chain (*MyHC I*) and downregulated expression of fast-twitch *MyHC IIb*, alongside increased ileal occludin expression. Moreover, the N3 treatment improved antioxidant capacity, as reflected by higher serum GSH and SOD levels and lower serum MDA content. In conclusion, decreasing dietary NE from 10.15 to 9.65 MJ/kg exerted no detrimental influence on growth performance yet effectively ameliorated meat quality, antioxidant status and intestinal barrier function in Tunchang Black pigs.

## Simple Summary

Proper dietary management is essential for regulating growth, meat quality and health status in pigs. The Tunchang Black pig is a native Chinese breed prized for its palatable meat, yet its optimal dietary energy requirement remains undefined. In the present study, four distinct dietary energy levels were assessed in juvenile pigs with an initial body weight of approximately 11 kg. The results revealed that the lowest dietary energy level suppressed growth rate and decreased carcass weight. By contrast, a moderate reduction in dietary energy below standard recommendations did not impair growth performance. This nutritional adjustment improved meat quality via decreasing undesirable saturated fatty acids and elevating concentrations of the beneficial mineral selenium. Enhanced antioxidant capacity was also observed in pork tissue, which maintains meat freshness. Furthermore, moderate energy restriction reinforced intestinal barrier function, which is vital for general health and nutrient absorption. Collectively, these findings indicate that moderately reduced dietary energy can yield healthier pork without retarding the growth of Tunchang Black pigs. This outcome facilitates efficient production of indigenous pig breeds and promotes the supply of high-quality pork products to consumers.

## 1. Introduction

Pork constitutes the predominant meat consumed by urban and rural residents across China, accounting for roughly two-thirds of the national total meat output [1]. In recent decades, alongside sustained improvements in living standards and evolving consumption preferences, the pork market has shifted its development focus from quantity supply to quality improvement [1,2]. Growing consumer demand has emerged for premium pork characterised by desirable marbling, balanced fatty acid composition and favourable sensory attributes [3,4,5]. This market trend presents new challenges to the pig production sector, particularly regarding the systematic enhancement of pork quality without compromising growth efficiency. Accordingly, establishing targeted nutritional modulation approaches to optimise meat quality has become a key research focus within animal nutrition and animal science disciplines.

Dietary energy level represents a core nutritional determinant regulating growth performance, muscle metabolism and meat quality in pigs. Adequate energy provision promotes muscle protein deposition and intramuscular fat accumulation, which further improves meat tenderness and juiciness, while either excessive or inadequate energy intake exerts negative impacts on meat quality [6,7,8]. Previous studies have confirmed that a moderate elevation in dietary energy can raise intramuscular fat content; in contrast, energy restriction accelerates muscle protein degradation, elevates drip loss and increases shear force values [9]. Apart from dietary energy, amino acid supply is also of vital importance. Sufficient essential amino acids, including lysine and methionine, not only act as substrates for muscle protein synthesis but also modulate muscle growth. As a methyl donor, methionine participates in creatine synthesis and further regulates muscular energy metabolism [10]. Furthermore, inosine monophosphate (IMP), the key substance responsible for meat umami flavour, exhibits accumulation patterns closely linked to muscle energy status, and its content is co-regulated by dietary energy and amino acid provision [11].

Skeletal muscle comprises different types of muscle fibres, and the proportion of these fibre types forms the structural basis for determining meat quality [12,13,14]. Based on the different isoforms of myosin heavy chain (*MyHC*), porcine skeletal muscle fibres are mainly classified into type I (slow-twitch oxidative), type IIa (fast-twitch oxidative-glycolytic), and type IIb (fast-twitch glycolytic) [15,16]. Type I fibres are abundant in mitochondria and myoglobin, which are positively correlated with meat redness value (a*) and oxidative stability, whereas type IIb fibres are predominantly glycolytic in metabolism and closely associated with problems such as pale meat colour and excessively rapid pH decline [17]. Previous studies have confirmed that dietary energy level can affect the expression of *MyHC* isoforms by regulating signalling pathways involved in muscle fibre type transformation, thereby modulating meat quality [3,18]. For example, a high-energy diet could upregulate the expression of *MyHC IIb* in the longissimus dorsi muscle of Ningxiang pigs, promoting the transformation of muscle towards a glycolytic type [6].

In addition to its direct regulation of muscle tissue, dietary energy level also indirectly modulates growth performance and meat quality by influencing intestinal health [19,20]. The intestine is not only the primary site for nutrient digestion and absorption but also the first line of defence against the invasion of external pathogens [21]. The integrity of intestinal barrier function is crucial for maintaining body homeostasis, preventing inflammatory responses, and ensuring feed conversion efficiency [22]. Studies have shown that insufficient energy intake can cause intestinal villus atrophy and crypt deepening, reducing the intestine’s absorptive capacity for nutrients, whereas excessive energy intake may induce intestinal oxidative stress and inflammatory responses, disrupt the structure of tight junction proteins, and increase intestinal permeability [23]. Tight junction proteins, such as Occludin, the Claudin family, and ZO-1, are key molecules maintaining the mechanical barrier function of the intestinal epithelium, and their expression levels directly reflect intestinal barrier integrity [24]. Therefore, when determining the appropriate dietary energy level, its impact on intestinal morphology, structure, and barrier function must be taken into account.

Oxidative stress constitutes a critical determinant of meat quality [25,26]. Pre- and post-slaughter stress responses induce the accumulation of reactive oxygen species in muscle tissue, triggering lipid peroxidation, protein oxidation and mitochondrial dysfunction, which compromises meat colour stability, elevates drip loss and accelerates lipid rancidity [27]. The Keap1-Nrf2-ARE signalling pathway serves as the core regulatory cascade for antioxidant enzyme expression and plays a vital role in the oxidative stress response [17,28,29]. Once activated, Nrf2 translocates to the nucleus and initiates the transcription of downstream antioxidant genes, including *HO-1* and *NQO1*, thereby enhancing cellular antioxidant capacity. Previous studies have demonstrated that moderate energy restriction activates the Nrf2 pathway and improves muscular antioxidant capability [30,31,32]. Furthermore, selenium, an essential trace element that acts as a prosthetic group for glutathione peroxidase (GSH-Px), is indispensable for endogenous antioxidant defence, and its tissue deposition efficiency is modulated by dietary energy levels [33,34,35].

The Tunchang Black pig is a high-quality meat-type pig breed crossbred and bred using the indigenous Hainan Tunchang pig as the maternal line and Duroc pig as the paternal line. It exhibits favourable tropical adaptability, crude feed tolerance and tender meat quality, occupying an important position in the pig industry of Hainan Province and its surrounding regions [36,37,38]. Nevertheless, systematic investigations into the optimal dietary net energy levels for growing Tunchang Black pigs remain scarce, and the effects of graded net energy levels on their growth performance, meat quality, muscle fibre characteristics, antioxidant capacity and intestinal health have not been documented. Current nutritional requirement standards for pigs are predominantly established based on foreign commercial breeds. Due to distinct differences in growth rate, fat deposition patterns and metabolic regulatory mechanisms, these recommended energy levels may not be suitable for indigenous pig breeds [39,40,41]. Therefore, optimising dietary net energy levels for Tunchang Black pigs is of great theoretical and practical significance for exploiting the meat quality potential of indigenous pig breeds and improving production efficiency.

Against the above background, this study aimed to systematically evaluate the effects of four graded dietary net energy levels (10.65, 10.15, 9.65 and 9.15 MJ/kg) on growth performance, carcass traits, meat quality, conventional nutrients, inosine monophosphate content, muscle fibre type composition, fatty acid profiles, muscle amino acid profiles, serum and muscular oxidative stress indices, and intestinal barrier function in 11–25 kg Tunchang Black pigs. The conventional recommended energy index is a dietary net energy level of 10.15 MJ/kg. We hypothesised that a moderate reduction in dietary net energy from the conventional recommended level (10.15 MJ/kg) to 9.65 MJ/kg would not impair growth performance but improve meat quality and intestinal health via optimising muscle fibre composition, regulating fatty acid profiles, enhancing muscular selenium deposition and reinforcing intestinal barrier function. The present findings provide a theoretical basis and empirical data for the development of precise nutritional strategies for indigenous pig breeds.

## 2. Materials and Methods

### 2.1. Animal Ethics Statement

The experimental design and procedures were authorised by the Animal Care and Use Committee of the Committee for Ethics in Animal Experimentation at the Tropical Crop Genetic Resource Research Institute, Chinese Academy of Tropical Agricultural Sciences (CATAS-2025012205).

### 2.2. Animals and Experimental Treatments

Forty-eight Tunchang Black pigs (half male and half female) with an average initial body weight of 11 kg were randomly allocated to four experimental groups, with 12 replicates per group. Pigs in the four groups were fed diets containing graded net energy (NE) levels of 10.65 MJ/kg (high-energy group, N1), 10.15 MJ/kg (control group, N2), 9.65 MJ/kg (low-energy group, N3) and 9.15 MJ/kg (very-low-energy group, N4), respectively. The basal diet for the N2 control group was formulated in accordance with the Chinese Swine Nutrient Requirements (GB/T39235-2020) [42]. The dietary composition and nutrient profiles are shown in Table 1. Each pen was fitted with a feeder and nipple drinker, allowing all pigs ad libitum access to feed and water throughout the trial. Feeders were inspected daily to avoid blockage and feed wastage, and feed consumption was recorded accordingly. At the end of the trial, final body weights were measured to calculate average daily gain (ADG) and feed-to-gain ratio (F/G). The entire experimental period lasted 42 days.

### 2.3. Sample Collection

At the end of the trial, all pigs were slaughtered for sample collection. Following the removal of the head, feet, tail and internal organs, carcass weight was recorded to calculate carcass yield. The longissimus dorsi (LD) muscle sampled from the sixth and seventh ribs on the right side of each carcass was either stored at −80 °C or immediately fixed in 4% paraformaldehyde.

### 2.4. Meat Quality

The redness (a*), yellowness (b*) and lightness (L*) values of the LD muscle were measured using a colour reader (KONICA MINOLTA, Japan). The pH values of the LD muscle at 45 min and 24 h post-slaughter were determined using a pH metre (TESTO, Germany). Fresh LD muscle samples were weighed (W1) and placed in sealed bags and stored at 4 °C for 24 h. The samples were subsequently reweighed (W2), and drip loss was calculated using the following formula: drip loss (%) = (W1 − W2)/W1 × 100%.

### 2.5. Determination of Conventional Nutrients and Inosinic Acid Content

In accordance with GB 5009.3-2016 [43], moisture content was determined by the direct drying method. Samples were dried at 101–105 °C to constant weight, and moisture content was calculated from the mass difference before and after drying. Following GB 5009.5-2016 [44], crude protein content was assayed via the Kjeldahl method. Samples underwent digestion, distillation and titration with standard acid solution, and crude protein values were calculated by multiplying total nitrogen content by a standard conversion factor. In line with GB 5009.6-2016 [45], crude fat content was measured using the Soxhlet extraction procedure. Samples were reflux-extracted with anhydrous ether or petroleum ether, and crude fat content was calculated according to the mass of extracted substances. As specified in GB 5009.4-2016 [46], ash content was determined by high-temperature incineration. Samples were carbonised and subsequently ignited in a muffle furnace at 550 ± 25 °C until constant weight was achieved, and crude ash content was calculated based on the mass of residual inorganic material.

Approximately 1 g of muscle tissue was accurately weighed, mixed with 20 mL of 5% perchloric acid and fully homogenised. After thorough blending, the mixture was incubated at 4 °C for 24 h, followed by centrifugation at 8000 rpm for 15 min to harvest the supernatant. The collected supernatant was filtered through a 0.22 μm membrane filter, and the pH of the filtrate was adjusted to 6.5. Inosinic acid concentration was quantified using an Agilent 1260 II (Agilent Technologies, Waldbronn, Germany) high-performance liquid chromatograph fitted with a diode array detector and a Thermo Fisher Scientific (Waltham, MA, USA) Acclaim 120 C18 column (4.6 mm × 250 mm, 5 μm).

### 2.6. Amino Acid Analysis

Freeze-dried longissimus dorsi muscle samples were trimmed to remove fascia using a scalpel and ophthalmic forceps and then minced using a meat grinder. The homogenised samples were evenly spread in pre-dried, pre-weighed Petri dishes and weighed accurately. Approximately 2 g of sample (accurate to 0.0001 g) was transferred into a 15 mL centrifuge tube, and a chloroform–methanol mixture (2:1, *v*/*v*) was added to fully submerge the tissue. The tube was shaken continuously until the muscle tissue turned pale. After balancing, samples were centrifuged at 3000 rpm and 4 °C for 5 min. Three distinct layers formed after centrifugation; the bottom layer was collected and filtered into a round-bottom flask. The flask was placed in an oven at 80 °C to remove all solvent, and the residual pale-yellow oil represented total sample lipids. Saponification and methyl esterification were conducted following the protocol described by Huang et al. [47]. Fatty acid profiles were subsequently determined via gas chromatography.

Fatty acid composition was analysed using an Agilent 7090B gas chromatograph fitted with a flame ionisation detector (FID) (Agilent Technologies, Waldbronn, Germany) and an Agilent J&W Cp-Sil 88 FAME column (100 m × 0.25 mm × 0.2 μm). A volume of 1 μL sample was injected in splitless mode via the front injector. The injector and detector temperatures were both maintained at 230 °C. Nitrogen was used as the carrier gas at a flow rate of 1.2 mL/min, and airflow was set at 300 mL/min. The oven temperature programme was set as follows: initial temperature held at 80 °C for 1 min, raised to 180 °C at 6 °C/min and maintained for 3 min, and then increased to 220 °C at 7 °C/min and held for a final 8 min.

### 2.7. Electronic Nose (E-Nose) Analysis

E-nose analysis was performed using a SuperNose-14 (ISENSO, Buffalo, NY, USA). Briefly, 2 g of LD muscle was added into a 20 mL headspace bottle [48]. All the samples were incubated at 80 °C for 15 min before analysis. The self-cleaning time and detection time were 120 s and 60 s, respectively. The sensor array used in the present study comprised 14 MOS sensors, specifically: s1 (sensitive to amines and ammonia), s2 (sensitive to sulfides and hydrogen sulfide), s3 (sensitive to hydrogen), s4 (sensitive to ethanol and other organic solvents), s6 (sensitive to biogas, natural gas and methane), s7 (sensitive to flammable gases), s8 (sensitive to volatile organic compounds), s9 (sensitive to natural gas and liquid gas), s10 (sensitive to flammable gases and liquid gas), s11 (sensitive to ethanol, alkanes, smoke and natural gas), s12 (sensitive to ethanol and organic solvents), s13 (sensitive to cooking odour and smoke), and s14 (sensitive to natural gas and methane).

### 2.8. Western Blotting

Proteins were extracted from the LD muscle and quantified using a bicinchoninic acid protein assay kit (Beyotime Biotechnology, Shanghai, China). A total of 10 μg of protein was separated by 10% sodium dodecyl sulfate–polyacrylamide gel electrophoresis and transferred to polyvinylidene fluoride membranes. The membranes were blocked with 5% nonfat milk for 2 h and incubated overnight at 4 °C with the following primary antibodies: β-Actin (CST, Danvers, MA, USA; 1:1000), myosin heavy chain I and IIb (MYHC I and MYHC IIb) (DSHB Iowa Ave, Iowa City, IA, USA; 1:1000), kelch-like ECH associated protein 1 (KEAP1) (Proteintech, Rosemont, IL, USA; 1:1000), heme oxygenase-1 (HO-1) (Proteintech, Rosemont, IL, USA; 1:1000), Nuclear factor erythroid 2-related factor 2 (NRF2) (Proteintech, Rosemont, IL, USA; 1:1000), Occludin (Proteintech, Rosemont, IL, USA; 1:1000), Claudin 1 (Proteintech, Rosemont, IL, USA; 1:1000) and Zonula occludens 1 (ZO1) (Proteintech, Rosemont, IL, USA; 1:1000). The membranes were washed and incubated with secondary antibody (Abclonal, Woburn, MA, USA; 1:5000). The density of bands was measured using Image J software (v1.54r) and normalised to the β-Actin.

### 2.9. Real-Time Quantitative RT-PCR

Total RNA was extracted from the longissimus dorsi (LD) muscle tissue using TRIzol reagent (Sangon Biotech Co., Ltd., Shanghai, China). Subsequently, complementary DNA (cDNA) synthesis was carried out strictly in accordance with the manufacturer’s protocol provided with the PrimeScript™ Reverse Transcription Reagent Kit (TaKaRa Biotechnology, Dalian, China). The primer sequences (including both forward and reverse primers) used in this study are listed in Table 2. The thermal cycling program for real-time quantitative polymerase chain reaction (RT-qPCR) was set as follows: an initial pre-denaturation/incubation step at 95 °C for 10 min, followed by 40 amplification cycles consisting of denaturation at 95 °C for 15 s, and combined annealing and extension at 60 °C for 30 s (a two-step protocol was employed). For data analysis, 18S ribosomal RNA (18S rRNA) was selected as the internal reference gene to normalise for variations in sample loading. The relative expression levels of the target genes were calculated using the 2^−∆∆Ct^ method (i.e., the comparative threshold cycle method).

### 2.10. Hematoxylin–Eosin (H&E) Staining

The tissues were embedded in paraffin, sectioned at a thickness of 5 μm, and subsequently stained with H&E. The morphology was observed using an inverted microscope (Leica dmi8, Leica Microsystems, Wetzlar, Germany). The diameters of LD muscle fibres, villus height, and crypt depth of intestine were analysed using Image J software (National Institutes of Health, Bethesda, MD, USA).

### 2.11. Fatty Acid Composition

Fatty acid composition in muscle was determined as described previously [24]. Lipids were extracted from muscle using the chloroform–methanol (1:1, *vol*/*vol*) procedure. Fatty acid methyl esters were detected using an Agilent 7090B gas chromatograph equipped with a flame ionization detector (FID) and an Agilent J&W Cp-Sil 88 GC column (100 m × 0.25 mm × 0.2 μm). The fatty acid content was calculated as a percentage of the total fatty acids.

### 2.12. Statistical Analysis

All experimental data are presented as the mean ± standard error of the mean (SEM). Raw data were initially collated using Microsoft Excel, followed by one-way analysis of variance (ANOVA) performed via SPSS Statistics (Version 27, IBM Corp., Armonk, NY, USA). Graph plotting was conducted using GraphPad Prism 8.0. Multiple comparisons among experimental groups were carried out using Duncan’s post hoc test. In all statistical analyses, a probability value of *p* < 0.05 was considered statistically significant, with differing superscript letters denoting significant differences between groups.

All experimental data are presented as the mean ± standard error of the mean (SEM). Raw data were initially collated using Microsoft Excel, followed by one-way analysis of variance (ANOVA) performed via SPSS Statistics (Version 27, IBM Corp., Armonk, NY, USA). In the statistical model, dietary net energy level was treated as a fixed factor, while pen was included as a random factor where applicable. Graph plotting was conducted using GraphPad Prism 8.0. Multiple comparisons among experimental groups were carried out using Duncan’s post hoc test. In all statistical analyses, a probability value of *p* < 0.05 was considered statistically significant, with differing superscript letters denoting significant differences between groups.

## 3. Results

### 3.1. Growth Performance and Carcass Traits

No significant differences (*p* > 0.05) were noted in the FW, ADG, ADFI, and F/G among the four groups (Table 3). However, the carcass weight and carcass yield were significantly decreased (*p* < 0.05) in the N4 group relative to the other three groups (Table 4). The backfat thickness was higher (*p* < 0.05) in the N3 group than in the other three groups (Table 4).

### 3.2. Meat Quality Traits

As shown in Table 5, the meat redness (a*) was reduced (*p* < 0.05) in the N1 group compared to the other three groups. No difference (*p* > 0.05) was observed in the meat yellowness (b*), brightness (L*), pH at 45 min and 24 h, drip loss, or shear force among the four groups.

### 3.3. Conventional Nutrients and Inosinic Acid

Regarding the muscle quality indicators shown in Table 6, there were no significant differences (*p* > 0.05) in the contents of moisture, dry matter (DM), and crude ash (Ash) among all treatment groups. The crude protein (CP) content in groups N3 and N4 was significantly higher than that in group N1 (*p* < 0.05). The ether extract (EE) content in groups N1 and N2 was significantly higher than that in group N4 (*p* < 0.05). The intramuscular fat (IMF) content was highest in group N2, which was significantly higher than that in groups N3 and N4 (*p* < 0.05). There was no significant difference (*p* > 0.05) in inosinic acid content among all groups.

### 3.4. The Levels of Amino Acids in Muscle

Regarding the amino acid composition shown in Table 7, most amino acid contents were not significantly affected by the treatment groups (*p* > 0.05). However, some amino acids showed significant differences among groups. Specifically, the contents of threonine (THR), isoleucine (ILE), lysine (LYS), cystine (CYS), and methionine (MET) differed significantly among groups (*p* < 0.05). In detail, the threonine content in group N3 was significantly higher than that in group N1 (*p* < 0.05); the isoleucine content in groups N3 and N4 was significantly higher than that in group N1 (*p* < 0.05); the lysine content was highest in group N3, significantly higher than that in the other groups (*p* < 0.05); the cystine content in groups N3 and N4 was significantly higher than that in groups N1 and N2 (*p* < 0.05); the methionine content in groups N3 and N4 was significantly higher than that in group N1 (*p* < 0.05). In addition, the total amino acid content (Sum) in groups N3 and N4 was significantly higher than that in group N1 (*p* < 0.05).

### 3.5. Fatty Acid Levels in Muscle

As shown in Table 8, the levels of saturated fatty acid (SFA) C10:0 and C12:0 in muscle were lower (*p* < 0.05) in the N1, N3 and N4 groups than in the N2 group. The level of unsaturated fatty acid (UFA) C18:2 in muscle was significantly increased (*p* < 0.05) in the N1 and N3 group compared to the N2 group.

### 3.6. E-Nose Analysis

**Figure 1:**
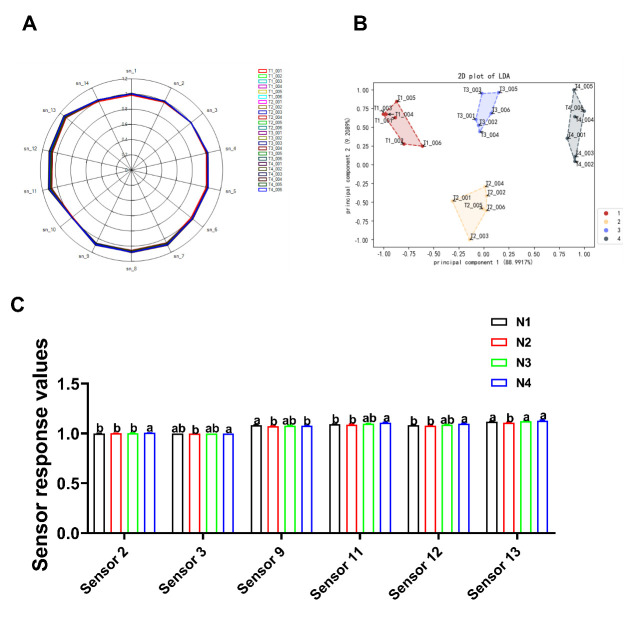
Data analysis for electronic nose. (**A**). Radar chart of the electronic nose response data. (**B**). Separation of the 4 groups in PCA space based on the data of electronic nose. (**C**). Electronic nose sensor responses to volatile substances.

As shown in Figure 1A, the total variance in contribution of the first two principal components was 98.19%, with the four groups clearly separated in the PCA plot. The response values of sensors 2 (sensitive to alkanes), 3 (sensitive to ozone), 11 (sensitive to volatile organic compounds), 12 (sensitive to alkanes, esters), and 13 (sensitive to long-chain alkanes) were higher (*p* < 0.05) in the N4 group than in the N2 group (Figure 1B,C). Moreover, the response value of sensor 13 (sensitive to long-chain alkanes) was higher (*p* < 0.05) in the N1 and N3 groups than in the N2 group (Figure 1B,C).

### 3.7. Muscle Fiber Diameter and Types

**Figure 2:**
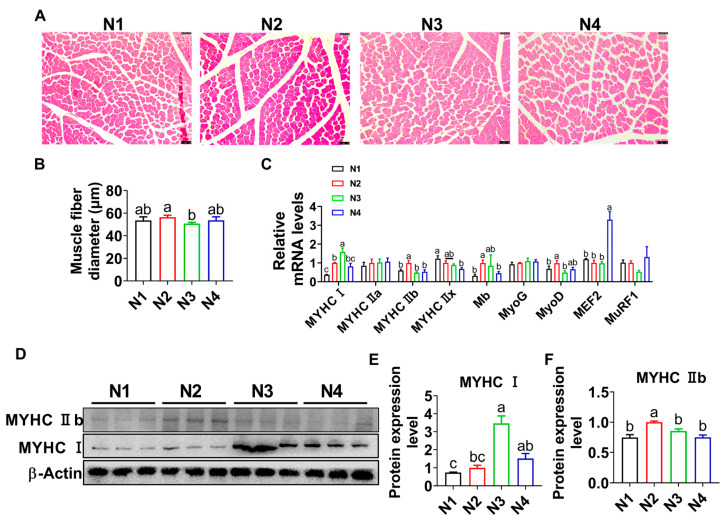
Effects of dietary NE level on muscle development. (**A**,**B**) H&E staining illustrating diameter of LD muscle fibres, bar = 50 μm. (**C**) Relative mRNA levels of genes associated with muscle development in LD muscle. (**D**–**F**) Protein expression levels of MYHC I and MYHC IIb in LD muscle. The results are shown as the mean ± SEM. Different letters indicate significant differences at *p* < 0.05.

The muscle diameter in the N2 group was significantly increased (*p* < 0.05) compared to the other three groups (Figure 2A,B). The mRNA expression level of MYHC1 was higher and that of MYHC IIB was lower (*p* < 0.05) in the N3 group than in the N2 group (Figure 2C). Compared to the N2 group, the protein expression level of MYHC I was increased (*p* < 0.05), while that of MYHC IIb was decreased (*p* < 0.05) in the N3 group (Figure 2D–F).

### 3.8. Oxidative Stress Levels in Serum and Muscle

**Figure 3:**
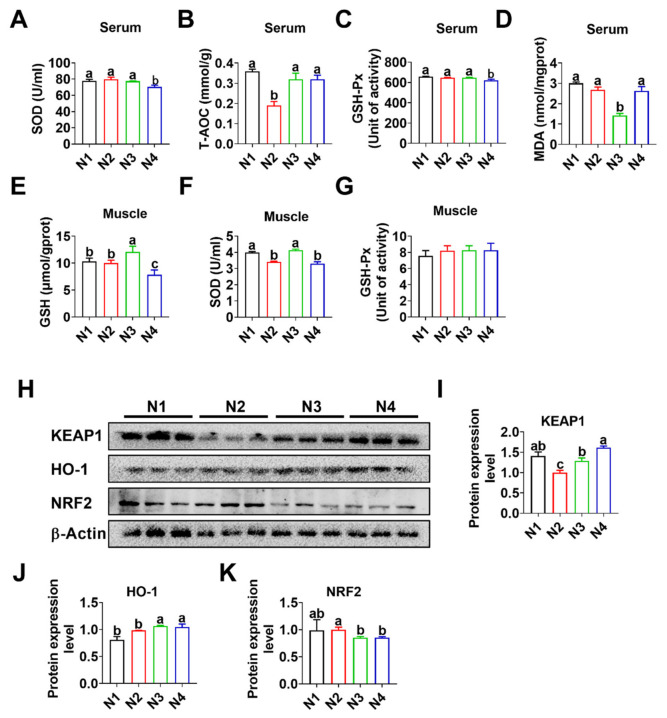
Oxidative stress levels in serum and muscle of Tunchang Black pigs. (**A**–**D**) Levels of SOD, T-AOC, GSH-Px and MDA in serum. (**E**–**G**) Levels of GSH, SOD and GSH-Px in LD muscle. (**H**–**K**) Protein levels of HO-1, NRF2, and KEAP1 in LD muscle. Different letters indicate significant differences at *p* < 0.05.

The contents of SOD and GSH-Px in serum were lower (*p* < 0.05) in the N4 group than in the other three groups (Figure 3A,C). Compared to the N2 group, the content of T-AOC was increased (*p* < 0.05), while that of MDA was decreased (*p* < 0.05) in the N3 group (Figure 3B,D). In the LD muscle, the contents of GSH and SOD were increased (*p* < 0.05) in the N3 group compared to the N2 group (Figure 3E,F), while no difference (*p* > 0.05) was observed in the content of GSH-Px among the four groups (Figure 3G). The protein expression levels of KEAP1 and HO-1 were higher (*p* < 0.05) in the N3 and N4 groups than in the N2 group (Figure 3H–J). Additionally, the protein expression level of NRF2 was reduced (*p* < 0.05) in the N3 and N4 groups compared to the N2 group (Figure 3H,K).

### 3.9. Intestinal Morphology and Barriers

**Figure 4:**
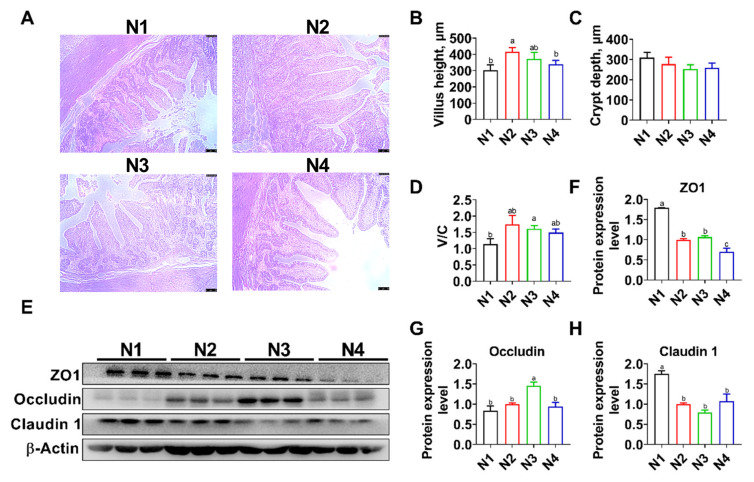
Effects of dietary NE level on intestinal development. H&E staining illustrating the villus height and crypt depth of ileum (**A**–**D**), bar = 50 μm. Protein levels of ZO-1, Occludin, and Claudin 1 in ileum (**E**–**H**). The results are shown as the mean ± SEM. Different letters indicate significant differences at *p* < 0.05.

In the ileum, the villus height was lower (*p* < 0.05) in the N1 and N4 groups than in the N2 group (Figure 4A,B). No difference (*p* > 0.05) was observed in the crypt depth among the four groups (Figure 4C). Compared to the N1 group, the V/C was increased (*p* < 0.05) in the N3 group (Figure 4D). However, the N1 group showed the highest (*p* < 0.05) expression levels of ZO-1 and Claudin 1 in the ileum (Figure 4E,F,H). The protein expression level of Occludin was increased (*p* < 0.05) in the N3 group compared to the N2 group (Figure 4E,G).

## 4. Discussion

Although the Tunchang Black pig is a valuable indigenous Chinese breed, a notable research gap remains concerning nutritional strategies for improving its meat quality characteristics, thereby limiting the consistent supply of high-quality pork from this breed. The present study demonstrated that reducing dietary net energy (NE) from 10.65 MJ/kg to 9.65 MJ/kg exerted no significant effects on final body weight, average daily feed intake, feed-to-gain ratio or carcass weight in Tunchang Black pigs. These results are consistent with previous findings indicating that moderate fluctuations in dietary energy intake have minimal impacts on such growth parameters in pigs [6]. However, pigs offered the lowest dietary NE level (9.15 MJ/kg) exhibited decreased average daily gain and carcass weight, indicating that this energy level fails to meet the metabolic requirements of growing Tunchang Black pigs. Such growth depression may be attributable to insufficient energy provision for protein deposition and muscle development, alongside a shift in energy partitioning towards bodily maintenance rather than growth. Collectively, these findings suggest that a dietary NE level of 9.65 MJ/kg is optimal for Tunchang Black pigs during the 11–25 kg growth stage.

Dietary energy level acts as a critical determinant of meat quality by modulating muscle metabolism and muscle fibre composition [49,50]. Meat redness (a*) values were lower in the N1 group (10.65 MJ/kg) relative to the other treatment groups, which may be closely linked to muscle fibre characteristics. The present study detected lower expression of slow-twitch MyHC I and higher expression of fast-twitch MyHC IIb in the N1 group. Slow-twitch oxidative (type I) muscle fibres are rich in myoglobin and mitochondria, which contribute to improved meat redness and enhanced oxidative stability [51]. In contrast, fast-twitch glycolytic (type IIb) fibres are associated with pale meat colour and accelerated post-mortem pH decline. Accordingly, the shift towards a glycolytic fibre phenotype in the high-energy group accounts for its reduced a* values [52]. By comparison, the N3 group (9.65 MJ/kg) maintained an intermediate muscle fibre composition, sustaining desirable redness while improving multiple meat quality attributes. Furthermore, reductions in C10:0, C12:0 and C18:2 were observed across the N1, N3 and N4 groups (Table 8), indicating that such alterations are not exclusive to moderate energy restriction [53,54]. Collectively, these findings demonstrate that, among the four graded dietary energy regimens evaluated, the 9.65 MJ/kg diet induced the most favourable modifications in fatty acid profiles and antioxidant capacity, despite partial beneficial responses being detected in the other treatment groups.

Consistent with the variations in fatty acid profiles, dietary net energy (NE) levels significantly altered the nutritional composition of muscle tissue. In this study, pigs in the N3 (9.65 MJ/kg) and N4 (9.15 MJ/kg) groups exhibited higher crude protein (CP) concentrations in the longissimus dorsi muscle than those in the N1 group (10.65 MJ/kg). Meanwhile, the N4 group had significantly lower crude fat (EE) and intramuscular fat (IMF) contents relative to the N1 and N2 groups. These results indicate that moderate dietary energy reduction shifts muscle nutrient deposition towards protein accretion rather than lipid accumulation, which supports previous studies demonstrating that energy restriction suppresses lipogenesis and enhances protein synthesis in growing pigs [7,55]. Notably, the N2 group (10.15 MJ/kg), corresponding to the conventional recommended dietary energy level, exhibited the greatest IMF content, suggesting that this energy regimen is optimal for marbling deposition in Tunchang Black pigs.

Furthermore, dietary NE levels significantly modulated the muscular amino acid profile. Compared with the N1 group, the N3 group exhibited significantly higher concentrations of threonine, isoleucine, lysine, cystine and methionine, alongside elevated total amino acid content. These essential amino acids are not only vital for protein synthesis but also act as precursors for flavour compounds and antioxidant substances [56,57]. The increased accumulation of lysine and methionine in the N3 group is particularly notable, as these amino acids are typically limiting in conventional pig diets and perform essential functions in muscle growth and antioxidant defence [58,59]. The greater total amino acid abundance, together with enhanced crude protein content, further confirms that a dietary NE level of 9.65 MJ/kg facilitates muscular protein deposition without impairing growth performance. Collectively, these findings indicate that appropriate reduction in dietary net energy improves the nutritional quality of pork by increasing muscular protein and essential amino acid contents in Tunchang Black pigs.

Oxidative stress is one of the primary factors responsible for deteriorated meat quality [27]. Accordingly, muscular oxidative stress biomarkers were determined in the present study. Compared with the N2 group, the N3 group exhibited increased muscular GSH and SOD contents and reduced serum MDA levels. These findings indicate that moderately lowered dietary energy alleviates muscular oxidative stress in pigs. Consistent with the present results, previous research has demonstrated that reducing dietary energy levels from 13.05 to 11.65 MJ/kg mitigates oxidative stress in weaned piglets [22]. HO-1, a member of the heat shock protein family, is implicated in cellular antioxidant defence and anti-apoptotic processes [60]. Existing studies have confirmed that the upregulation of HO-1 suppresses reactive oxygen species generation and enhances antioxidant responses, thereby attenuating stress-induced cellular damage [61,62]. The KEAP1 signalling pathway is essential for the defence against oxidative stress [63,64]. In this study, the N3 group showed an improved oxidative status, characterised by higher GSH and SOD concentrations and lower MDA levels, along with elevated HO-1 protein expression. Notably, Keap1 protein levels were increased, while total Nrf2 levels were decreased in the N3 group. Given that Keap1 acts as a negative regulator of Nrf2, this expression pattern suggests that the canonical Keap1–Nrf2 pathway may not be the predominant mechanism driving HO-1 upregulation under the present experimental conditions. Nrf2-independent pathways (e.g., MAPK and AP-1) or post-translational modulation of Nrf2 activity (e.g., nuclear translocation) may contribute to this regulatory effect [65]. As nuclear Nrf2 levels and Nrf2 phosphorylation status were not assessed in the current study, the precise underlying mechanism remains to be clarified.

Intestinal health is essential for maintaining optimal growth and development in pigs [66,67,68]. Previous studies have shown that both insufficient and excessive energy intake impairs intestinal development in pigs, resulting in reduced nutrient utilisation and growth performance [22,69]. In the present study, pigs fed diets with either low (9.15 MJ/kg; N4) or high (10.65 MJ/kg; N1) net energy (NE) levels exhibited significantly decreased jejunal villus height compared with the N2 control group (10.15 MJ/kg). These observations are consistent with previous studies reporting that both energy-deficient and energy-excessive diets disrupt intestinal morphology, which may be attributed to altered cellular proliferation, oxidative stress and inflammatory responses [23,61,70]. Notably, distinct response patterns were detected among different tight junction proteins: the N3 group showed upregulation of Occludin, while the N1 group exhibited increased ZO-1 and Claudin 1 expression. Nevertheless, direct measurements of intestinal permeability and inflammatory biomarkers are required to verify the functional implications of these alterations in tight junction protein abundance. These differential responses indicate energy-dependent and target-specific regulation rather than a universal improvement in intestinal barrier function induced by a single dietary energy level. The functional outcomes of such varied expression profiles remain to be elucidated. Overall, a moderate dietary energy supply better preserves intestinal epithelial integrity, which is critical for inhibiting pathogen translocation and improving nutrient absorption. The enhanced intestinal development observed in the N3 group may be mediated by optimised metabolic signalling pathways, such as the mTOR pathway, which modulates energy homeostasis and intestinal epithelial barrier function [24,71]. Collectively, these findings demonstrate that a dietary NE level of 9.65 MJ/kg optimally supports intestinal development in growing Tunchang Black pigs.

Several limitations of the present study should be acknowledged. Firstly, the 42-day trial only covered the early growth phase (11–25 kg), which may be insufficient to fully assess the long-term effects of dietary net energy on growth performance and carcass characteristics, including backfat thickness and intramuscular fat deposition, traits that develop progressively throughout the growth-finishing period. Longer trials extending to the finishing stage are, therefore, warranted. Secondly, key meat quality indicators, including drip loss and shear force, showed no significant treatment differences, which may be attributable to the relatively short feeding duration or the unique genetic characteristics of Tunchang Black pigs. Thirdly, direct consumer sensory evaluation was not performed; future research should incorporate consumer panel assessments to correlate variations in muscle composition with meat palatability. Fourthly, the graded net energy levels in this experiment were achieved by adjusting soybean oil inclusion rates, which decreased from 7.33% in the N1 group to 0% in the N4 group. This design limitation prevents the differentiation of independent effects derived from dietary net energy density and soybean oil supplementation per se. Future studies adopting isocaloric formulations with distinct energy sources or factorial experimental designs are required to separate the impacts of energy density from those of fatty acid or antioxidant additives. Fifth, we did not include sex as a factor in the main analysis. However, we acknowledge that sex may influence the examined traits, and future studies with larger sample sizes should consider testing for sex effects and sex-by-energy interactions. Finally, the optimal dietary NE level identified in this study (9.65 MJ/kg) is specific to Tunchang Black pigs within the 11–25 kg growth stage, and further validation is essential before extrapolation to other indigenous pig breeds or alternative growth phases.

## 5. Conclusions

This study demonstrates that reducing dietary net energy from 10.65 MJ/kg to 9.65 MJ/kg does not compromise the growth performance of Tunchang Black pigs whilst significantly improving their meat quality and intestinal health. Specifically, the 9.65 MJ/kg dietary regimen reduces the concentrations of saturated fatty acids (C10:0 and C12:0), elevates selenium accumulation, enhances antioxidant capacity (via increased GSH and SOD levels and reduced MDA content), and promotes a favourable shift towards oxidative muscle fibre phenotypes (the upregulation of MyHC I and downregulation of MyHC IIb). These modifications confer tangible consumer benefits, including reduced dietary intake of undesirable saturated fatty acids, strengthened endogenous antioxidant defence, and potentially improved meat colour stability and freshness. Furthermore, pigs in the N3 group exhibited enhanced ileal Occludin expression, indicating partial improvements in intestinal barrier integrity. Nevertheless, other tight junction proteins (ZO-1 and Claudin 1) were upregulated in the high-energy group, suggesting complex, protein-specific regulatory responses to dietary energy levels.

## Figures and Tables

**Table 1:** Ingredients and nutrient composition of diets (on air-dry basis).

Ingredients	N1	N2	N3	N4
Corn, %	47.71	50.86	53.9	57.03
Soybean meal, %	14.94	14.26	13.62	12.95
Wheat bran, %	27.65	27.65	27.65	27.65
Soybean oil, %	7.33	4.86	2.45	0
Stone powder, %	1.50	1.50	1.50	1.50
NaCl, %	0.29	0.29	0.29	0.29
L-lysine, %	0.01	0.02	0.03	0.03
DL-methionine, %	0.02	0.01	0.01	0
Compound premix ^1^, %	0.25	0.25	0.25	0.25
Mold inhibitor, %	0.10	0.10	0.10	0.10
Complex enzymes, %	0.10	0.10	0.10	0.10
Compound acidifier, %	0.10	0.10	0.10	0.10
Total, %	100.00	100.00	100.00	100.00
Nutrient components				
Net energy ^2^/(MJ/kg)	10.65	10.15	9.65	9.15
Crude protein ^3^, %	14.99	14.99	14.99	14.99
Crude fat ^3^, %	10.28	7.97	5.71	3.41
Calcium ^3^, %	0.62	0.62	0.62	0.61
Phosphorus ^3^, %	0.53	0.54	0.54	0.55
Lysine ^2^, %	0.68	0.68	0.68	0.68
Methionine + Cysteine ^2^, %	0.54	0.54	0.54	0.54

^1^ Vitamin premix provides the following per kg of diet: Vitamin A (retinol acetate), 12,000 IU; Vitamin D_3,_ 4500 IU; Vitamin E (DL-a-tocopherol acetate), 20 IU; Vitamin K_3_, 2 mg; Vitamin B_1_, 2 mg; Vitamin B_2_, 8 mg; Vitamin B_6_, 4 mg; Vitamin B_12_, 0.021 mg; d-pantopantoic acid, 1 mg; folic acid, 1 mg; d-biotin, 0.1 mg, Se as Na_2_SeO_3_, 0.33 mg; I as KIO_3_, 0.35 mg; Cu as CuSO_4_·5H_2_O, 12 mg; Mn as MnSO_4_·H_2_O, 60 mg; Fe as FeSO_4_·7H_2_O, 80 mg; Zn as ZnSO_4_·7H_2_O, 75 mg. ^2^ Calculated value. ^3^ Analysed content.

**Table 2:** Primers used for real-time PCR.

Names	Accession No.	Primer Sequences (5′ → 3′)	Product (bp)
*MyHC I*	U75316	F1: AGCCTCTTTCTTCTCCCAGGGACATTC	384
		R1: ATCCAGGCTGCGTAACGCTCTTTGAGGTTGTA	
*MyHC IIa*	AB025260	F2: CACTTGCTAAGAGGGACCTCTGAGTTCA	375
		R2: ATCCAGGCTGCGTAACGCTCTTTGAGGTTGTA	
*MyHC IIx*	AB025262	F3: CTTTCCTCATAAAGCTTCAAGTTCTGCC	429
		R3: ATCCAGGCTGCGTAACGCTCTTTGAGGTTGTA	
*MyHC IIb*	AB025261	F4: CATCTGGTAACATAAGAGGTACATCTAG	398
		R4: ATCCAGGCTGCGTAACGCTCTTTGAGGTTGTA	
*18S rRNA*	NR_046261.1	F: CCCACGGAATCGAGAAAGAG	117
		R: TTGACGGAAGGGCACCA	

*MyHC I*: myosin heavy chain I; *MyHC IIa*: myosin heavy chain 2a; *MyHC IIx*: myosin heavy chain 2x; *MyHC IIb*: myosin heavy chain 2b.

**Table 3:** Effects of dietary NE level on growth performance of Tunchang Black pigs.

Items	N1	N2	N3	N4	*p*-Value
Initial body weight, kg	11.63 ± 0.51	11.63 ± 0.54	11.61 ± 0.50	11.63 ± 0.46	0.997
Final body weight, kg	22.71 ± 1.92	22.69 ± 2.31	25.98 ± 1.13	19.66 ± 1.27	0.121
ADG, g/d	272.14 ± 39.81	275.24 ± 37.74	333.57 ± 32.58	198.10 ± 33.01	0.113
ADFI, kg/d	1.03 ± 0.05	0.99 ± 0.04	1.09 ± 0.15	0.90 ± 0.05	0.472
F/G, kg/kg	4.15 ± 0.65	3.11 ± 0.55	3.51 ± 0.72	5.13 ± 0.95	0.274

N1 (*n* = 12), 10.65 MJ/kg; N2 (*n* = 12), 10.15 MJ/kg, control group; N3 (*n* = 12), 9.65 MJ/kg; and N4 (*n* = 12), 9.15 MJ/kg.

**Table 4:** Effects of dietary NE level on carcass traits of Tunchang Black pigs.

Items	N1 (*n* = 12)	N2 (*n* = 12)	N3 (*n* = 12)	N4 (*n* = 12)	*p*-Value
Carcass weight, kg	14.49 ± 1.25 ^ab^	14.80 ± 1.52 ^ab^	16.77 ± 0.94 ^a^	11.39 ± 0.81 ^b^	0.041
Carcass yield, %	63.77 ± 0.92 ^a^	65.20 ± 2.48 ^a^	64.20 ± 1.02 ^a^	58.00 ± 1.58 ^b^	0.032
Backfat thickness, mm	18.92 ± 1.98 ^b^	19.74 ± 1.50 ^b^	24.46 ± 1.43 ^a^	16.37 ± 1.20 ^b^	0.023

^a,b^ Means with different superscripts within a row differ (*p* < 0.05). N1 (*n* = 12), 10.65 MJ/kg; N2 (*n* = 12), 10.15 MJ/kg, control group; N3 (*n* = 12), 9.65 MJ/kg; and N4 (*n* = 12), 9.15 MJ/kg.

**Table 5:** Effects of dietary NE level on meat quality of Tunchang Black pigs.

Items	N1 (*n* = 12)	N2 (*n* = 12)	N3 (*n* = 12)	N4 (*n* = 12)	*p*-Value
Redness (a*)	2.14 ± 0.34 ^b^	11.64 ± 3.04 ^a^	9.92 ± 2.14 ^a^	8.28 ± 2.57 ^ab^	0.041
Yellowness (b*)	7.54 ± 0.55	9.66 ± 0.52	8.34 ± 0.85	7.10 ± 0.70	0.073
Brightness (L*)	45.08 ± 1.41	42.18 ± 1.66	42.00 ± 2.47	39.96 ± 1.12	0.263
Drip loss, %	5.11 ± 0.53	6.43 ± 0.75	5.16 ± 1.06	6.63 ± 0.92	0.454
pH_45min_	5.85 ± 0.09	6.07 ± 0.14	6.12 ± 0.07	5.98 ± 0.06	0.252
pH_24h_	5.26 ± 0.02	5.27 ± 0.06	5.19 ± 0.04	5.32 ± 0.09	0.491
Shear force, N	55.54 ± 5.26	59.72 ± 3.24	62.68 ± 4.26	59.58 ± 8.56	0.852

^a,b^ Means with different superscripts within a row differ (*p* < 0.05). N1 (*n* = 12), 10.65 MJ/kg; N2 (*n* = 12), 10.15 MJ/kg, control group; N3 (*n* = 12), 9.65 MJ/kg; and N4 (*n* = 12), 9.15 MJ/kg.

**Table 6:** Levels of conventional nutrients and inosinic acid in the muscle of Tunchang pigs.

Items	N1	N2	N3	N4	*p*-Value
Moisture (%)	68.52 ± 2.73	68.52 ± 2.73	68.85 ± 3.06	68.98 ± 2.76	0.999
DM (%)	31.48 ± 1.02	31.32 ± 1.43	31.15 ± 1.02	31.02 ± 1.43	0.994
Ash (%)	1.04 ± 0.03	1.03 ± 0.03	1.03 ± 0.04	1.02 ± 0.02	0.958
CP (%)	16.85 ± 0.39 ^b^	17.85 ± 0.78 ^ab^	19.25 ± 0.66 ^a^	19.35 ± 0.65 ^a^	0.034
EE (%)	11.45 ± 0.25 ^a^	11.48 ± 0.36 ^a^	10.89 ± 0.41 ^ab^	10.23 ± 0.08 ^b^	0.028
IMF (%)	8.84 ± 0.23 ^ab^	9.37 ± 0.23 ^a^	8.67 ± 0.26 ^b^	8.34 ± 0.06 ^b^	0.018
Inosinic acid (mg/g)	1.28 ± 0.27	1.26 ± 0.07	1.26 ± 0.09	1.19 ± 0.06	0.960

^a,b^ Means with different superscripts within a row differ (*p* < 0.05). N1 (*n* = 12), 10.65 MJ/kg; N2 (*n* = 12), 10.15 MJ/kg, control group; N3 (*n* = 12), 9.65 MJ/kg; and N4 (*n* = 12), 9.15 MJ/kg.

**Table 7:** Levels of various amino acids in the muscle of Tunchang pigs.

Items (mg/g)	N1	N2	N3	N4	*p*-Value
ASP	3.42 ± 0.19	3.55 ± 0.21	3.65 ± 0.18	3.55 ± 0.19	0.871
THR	4.74 ± 0.20 ^b^	5.20 ± 0.18 ^ab^	5.71 ± 0.13 ^a^	5.35 ± 0.22 ^a^	0.013
SER	13.75 ± 0.63	13.95 ± 0.59	14.20 ± 0.63	14.15 ± 0.59	0.952
GLU	15.85 ± 0.59	16.30 ± 0.59	15.82 ± 0.33	16.55 ± 0.59	0.729
GLY	16.20 ± 0.76	16.25 ± 0.71	15.85 ± 0.72	15.75 ± 0.71	0.948
ALA	55.30 ± 2.24	56.50 ± 2.13	55.43 ± 1.15	56.80 ± 1.84	0.923
VAL	5.55 ± 0.18	5.52 ± 0.22	5.58 ± 0.18	5.55 ± 0.22	0.998
ILE	3.99 ± 0.18 ^c^	4.09 ± 0.24 ^bc^	4.81 ± 0.18 ^a^	4.59 ± 0.14 ^ab^	0.017
TYR	5.52 ± 0.18	5.55 ± 0.22	5.62 ± 0.22	5.58 ± 0.21	0.989
PHE	5.65 ± 0.20	5.70 ± 0.22	5.78 ± 0.20	5.72 ± 0.18	0.975
HIS	1.45 ± 0.13	1.48 ± 0.22	1.73 ± 0.15	1.50 ± 0.18	0.670
LYS	9.68 ± 0.11 ^b^	10.25 ± 0.30 ^b^	11.18 ± 0.24 ^a^	10.35 ± 0.39 ^b^	0.010
ARG	9.70 ± 0.43	10.30 ± 0.53	10.95 ± 0.38	10.80 ± 0.49	0.244
PRO	14.35 ± 0.25	14.25 ± 0.26	13.75 ± 0.28	13.65 ± 0.22	0.155
CYS	52.40 ± 0.54 ^b^	52.60 ± 0.14 ^b^	59.97 ± 2.08 ^a^	57.80 ± 2.27 ^a^	0.005
MET	1.90 ± 0.15	2.30 ± 0.10	2.48 ± 0.14	2.38 ± 0.08	0.013
Sum	220.05 ± 2.30 ^c^	223.79 ± 1.61 ^bc^	232.51 ± 1.98 ^a^	229.57 ± 2.98 ^ab^	0.004

^a–c^ Means with different superscripts within a row differ (*p* < 0.05). N1 (*n* = 12), 10.65 MJ/kg; N2 (*n* = 12), 10.15 MJ/kg, control group; N3 (*n* = 12), 9.65 MJ/kg; and N4 (*n* = 12), 9.15 MJ/kg.

**Table 8:** Fatty acid composition in muscle (%).

Items	N1	N2	N3	N4	*p*-Value
C10:0	4.17 ± 0.18 ^b^	5.57 ± 0.20 ^a^	4.69 ± 0.58 ^ab^	4.37 ± 0.21 ^b^	0.039
C11:0	0.54 ± 0.14	0.97 ± 0.18	0.76 ± 0.15	0.77 ± 0.14	0.313
C12:0	4.14 ± 0.44 ^b^	5.37 ± 0.08 ^a^	4.63 ± 0.22 ^ab^	4.45 ± 0.22 ^b^	0.029
C13:0	1.59 ± 0.22	1.67 ± 0.21	1.65 ± 0.18	1.89 ± 0.20	0.761
C14:0	6.73 ± 0.20	6.68 ± 0.35	6.91 ± 0.35	6.85 ± 0.31	0.947
C14:1	1.06 ± 0.18	1.12 ± 0.14	1.12 ± 0.22	1.19 ± 0.22	0.974
C15:0	0.96 ± 0.22	1.29 ± 0.18	1.28 ± 0.18	1.29 ± 0.18	0.564
C15:1	1.13 ± 0.22	1.12 ± 0.14	1.34 ± 0.20	1.19 ± 0.19	0.838
C16:0	17.23 ± 0.28	16.56 ± 0.55	17.07 ± 0.24	17.10 ± 0.46	0.662
C17:1	1.73 ± 0.22	1.53 ± 0.18	1.39 ± 0.22	1.65 ± 0.19	0.677
C18:0	20.59 ± 0.61	19.85 ± 1.02	20.17 ± 0.61	19.31 ± 0.69	0.677
C18:1	4.54 ± 0.22	4.33 ± 0.21	4.17 ± 0.21	4.19 ± 0.19	0.583
C18:2	35.59 ± 0.48 ^a^	32.27 ± 1.31 ^b^	34.95 ± 0.36 ^a^	35.78 ± 0.56 ^a^	0.015
Total SFA	55.95 ± 0.62	57.90 ± 0.94	57.09 ± 0.77	56.00 ± 1.48	0.450
Total UFA	44.05 ± 0.69	42.10 ± 1.93	42.91 ± 0.59	44.00 ± 0.61	0.444

1 SFA, saturated fatty acid. Total SFA = C10:0 + C11:0 + C12:0 + C13:0 + C14:0 + C15:0 + C16:0 + C18:0. 2 UFA, unsaturated fatty acid. Total UFA = C14:1 + C15:1 + C17:1 + C18:1 + C18:2. ^a,b^ Means with different superscripts within a row differ (*p* < 0.05). N1 (*n* = 12), 10.65 MJ/kg; N2 (*n* = 12), 10.15 MJ/kg, control group; N3 (*n* = 12), 9.65 MJ/kg; and N4 (*n* = 12), 9.15 MJ/kg.

## Data Availability

The data presented in this study are available on request from the corresponding author due to limited by the experimental research.

## Abbreviations

The following abbreviations are used in this manuscript:

ADG	Average daily gain
ADFI	Average daily feed intake
ANOVA	Analysis of variance
CP	Crude protein
DM	Dry matter
EE	Ether extract
E-nose	Electronic nose
F/G	Feed-to-gain ratio
GSH	Glutathione
GSH-Px	Glutathione peroxidase
H&E	Hematoxylin and eosin
*HO-1*	*Heme oxygenase-1*
*NQO1*	*NAD(P)H quinone dehydrogenase 1*
IMF	Intramuscular fat
KEAP1	Kelch-like ECH associated protein 1
LD	Longissimus dorsi
MDA	Malondialdehyde
MyHC	Myosin heavy chain
NE	Net energy
NRF2	Nuclear factor erythroid 2-related factor 2
PCA	Principal component analysis
SFA	Saturated fatty acid
SOD	Superoxide dismutase
T-AOC	Total antioxidant capacity
UFA	Unsaturated fatty acid
ZO-1	Zonula occludens-1

## References

[1] Zhang H., Shi Z., Zhou H., Hu X. (2023). Pork Consumption Patterns among Rural Residents in China: A Regional and Cultural Perspective (2000–2020). Agriculture.

[2] Liang Y., Cheng Y., Xu Y., Hua G., Zheng Z., Li H., Han L. (2022). Consumer Preferences for Animal Welfare in China: Optimization of Pork Production-Marketing Chains. Animals.

[3] Yang C., Wang W., Tang X., Huang R., Li F., Su W., Yin Y., Wen C., Liu J. (2022). Comparison of the Meat Quality and Fatty Acid Profile of Muscles in Finishing Xiangcun Black Pigs Fed Varied Dietary Energy Levels. Anim. Nutr..

[4] Tretola M., Mazzoleni S., Silacci P., Dubois S., Proserpio C., Pagliarini E., Bernardi C.E.M., Pinotti L., Bee G. (2024). Sustainable Pig Diets: Partial Grain Replacement with Former Food Products and Its Impact on Meat Quality. J. Anim. Sci..

[5] Mulders M.D.G.H., Grunert K.G., Pedersen S., Brunsø K., Zhou Y. (2024). Pleasure, Quality or Status? An Analysis of Drivers of Purchase of Fresh Pork in China. Front. Anim. Sci..

[6] Chen J., Chen F., Lin X., Wang Y., He J., Zhao Y. (2020). Effect of Excessive or Restrictive Energy on Growth Performance, Meat Quality, and Intramuscular Fat Deposition in Finishing Ningxiang Pigs. Animals.

[7] Yan E., Guo J., Yin J. (2023). Nutritional Regulation of Skeletal Muscle Energy Metabolism, Lipid Accumulation and Meat Quality in Pigs. Anim. Nutr..

[8] Fazarinc G., Vrecl M., Poklukar K., Škrlep M., Batorek-Lukač N., Brankovič J., Tomažin U., Čandek-Potokar M. (2020). Expression of Myosin Heavy Chain and Some Energy Metabolism-Related Genes in the Longissimus Dorsi Muscle of Krškopolje Pigs: Effect of the Production System. Front. Vet. Sci..

[9] Zeng Z., Yu B., Mao X., Chen D. (2012). Effects of Dietary Digestible Energy Concentration on Growth, Meat Quality, and PPARγ Gene Expression in Muscle and Adipose Tissues of Rongchang Piglets. Meat Sci..

[10] Rezaei R., Wang W., Wu Z., Dai Z., Wang J., Wu G. (2013). Biochemical and Physiological Bases for Utilization of Dietary Amino Acids by Young Pigs. J. Anim. Sci. Biotechnol..

[11] Ye F., Deng Z.-D., Liu K.-Y., Yao X.-M., Zheng W.-X., Yin Q., Hai X., Gan J.-K., Zhang Z.-F., Ma Z. (2024). Integrative Analysis of the Transcriptome, Proteomics and Metabolomics Reveals Key Genes Involved in the Regulation of Breast Muscle Metabolites in Capons. BMC Genom..

[12] Liu Y., Zhang M., Shan Y., Ji G., Ju X., Tu Y., Sheng Z., Xie J., Zou J., Shu J. (2020). miRNA-mRNA Network Regulation in the Skeletal Muscle Fiber Phenotype of Chickens Revealed by Integrated Analysis of miRNAome and Transcriptome. Sci. Rep..

[13] Feng Z., Wang X., Zhou Q., Liu Y., Xu R., Liang Z., Zhang C., Liu X., Zhao Y., Chen Y. (2025). Integrated Proteomics and Transcriptomics Analysis of Dynamic Changes in Muscle Fiber Types in Different Regions of Porcine Skeletal Muscle. Adv. Biotechnol..

[14] Mo M., Zhang Z., Wang X., Shen W., Zhang L., Lin S. (2023). Molecular Mechanisms Underlying the Impact of Muscle Fiber Types on Meat Quality in Livestock and Poultry. Front. Vet. Sci..

[15] Shi L.-L., Sun Y.-T., Sun J.-N., Yue J., Chen W., Meng K., Chen L., Hu C.-Q., Chen R., Sun D.-S. (2025). VEGF-B-Mediated Myofiber Types Involved in High-Fat Diet-Induced Hyperglycemia through PKA-NFATs Signaling Pathway. Stem Cell Res. Ther..

[16] Meissner J.D., Freund R., Krone D., Umeda P.K., Chang K.-C., Gros G., Scheibe R.J. (2011). Extracellular Signal-Regulated Kinase 1/2-Mediated Phosphorylation of P300 Enhances Myosin Heavy Chain I/Beta Gene Expression via Acetylation of Nuclear Factor of Activated T Cells C1. Nucleic Acids Res..

[17] Ma X., Guo X., La Y., Wu X., Chu M., Bao P., Yan P., Liang C. (2023). Integrative Analysis of Proteomics and Transcriptomics of Longissimus Dorsi with Different Feeding Systems in Yaks. Foods.

[18] Wang Y., Zhang D., Liu Y. (2024). Research Progress on the Regulating Factors of Muscle Fiber Heterogeneity in Livestock: A Review. Animals.

[19] Wang Z., Geng C., Zhang J., Zeng X., Wang X., Zhang C., Zhang W., Wang Q., Yang H., Yin Y. (2025). Effects of Dietary Digestible Energy Levels on Growth Performance, Intestinal Function, Carcass Traits, Meat Quality and Blood Biochemical Parameters of Ningxiang Pigs. Anim. Nutr..

[20] Zhang D., Chu M., Ge Q., Yan P., Bao P., Ma X., Guo X., Liang C., Wu X. (2024). Effects of Dietary Energy Levels on Growth Performance, Serum Metabolites, and Meat Quality of Jersey Cattle-Yaks. Foods.

[21] Wang W., Wang Y., Huang P., Zhou J., Tan G., Zeng J., Liu W. (2024). Mosla Chinensis Extract Enhances Growth Performance, Antioxidant Capacity, and Intestinal Health in Broilers by Modulating Gut Microbiota. Microorganisms.

[22] Adebowale T., Jiang Q., Yao K. (2024). Dietary Fat and High Energy Density Diet: Influence on Intestinal Health, Oxidative Stress and Performance of Weaned Piglets. J. Anim. Physiol. Anim. Nutr..

[23] Wang Y., Yang C., Elsheikh N.A.H., Li C., Yang F., Wang G., Li L. (2019). HO-1 Reduces Heat Stress-Induced Apoptosis in Bovine Granulosa Cells by Suppressing Oxidative Stress. Aging.

[24] Li M.-X., Li M.-Y., Lei J.-X., Wu Y.-Z., Li Z.-H., Chen L.-M., Zhou C.-L., Su J.-Y., Huang G.-X., Huang X.-Q. (2022). Huangqin Decoction Ameliorates DSS-Induced Ulcerative Colitis: Role of Gut Microbiota and Amino Acid Metabolism, mTOR Pathway and Intestinal Epithelial Barrier. Phytomed. Int. J. Phytother. Phytopharm..

[25] Składanowska-Baryza J., Ludwiczak A., Pruszyńska-Oszmałek E., Kołodziejski P., Stanisz M. (2020). Effect of Two Different Stunning Methods on the Quality Traits of Rabbit Meat. Animals.

[26] Chen K., Lv J., Luo Z., Liu Z., Cen M., Li B., Ou J., Zhang H. (2025). The Effect of Amylase, Chromium Propionate and Their Combination Supplementation on Growth Performance, Carcass Traits, Serum Parameters, Antioxidant Capacity and Intestinal Health in Yellow Feathered Broilers. Poult. Sci..

[27] Chen Z., Xing T., Li J., Zhang L., Jiang Y., Gao F. (2022). Oxidative Stress Impairs the Meat Quality of Broiler by Damaging Mitochondrial Function, Affecting Calcium Metabolism and Leading to Ferroptosis. Anim. Biosci..

[28] Zhao Y., Zhuang Y., Shi J., Fan H., Lv Q., Guo X. (2025). Cathepsin B Induces Kidney Diseases through Different Types of Programmed Cell Death. Front. Immunol..

[29] Mj K., Hg K., Sb J., Jh Y., Ps J., Bw S., Su K., Sk C., Bs S. (2024). The Antioxidant Betulinic Acid Enhances Porcine Oocyte Maturation through Nrf2/Keap1 Signaling Pathway Modulation. PLoS ONE.

[30] Xie Y., Jin Y., Li S., Shen B., Ma L., Zuo L., Gao Y., Yang G. (2023). Leonurine Alleviates Cognitive Dysfunction and Reduces Oxidative Stress by Activating Nrf-2 Pathway in Alzheimer’s Disease Mouse Model. Neuropsychiatr. Dis. Treat..

[31] Qin S., Zhu J., Qin S., Hu X., Li J., Tang D., Shi Z. (2026). Selenium Yeast Improves Egg Production Performance by Increasing Antioxidant Capacity, Reproductive Hormone Levels and Yolk Precursor Synthesis in Late Peak Laying Hens. Poult. Sci..

[32] Gao S., Heng N., Liu F., Guo Y., Chen Y., Wang L., Ni H., Sheng X., Wang X., Xing K. (2021). Natural Astaxanthin Enhanced Antioxidant Capacity and Improved Semen Quality through the MAPK/Nrf2 Pathway in Aging Layer Breeder Roosters. J. Anim. Sci. Biotechnol..

[33] Jing J., He Y., Liu Y., Tang J., Wang L., Jia G., Liu G., Chen X., Tian G., Cai J. (2023). Selenoproteins Synergistically Protect Porcine Skeletal Muscle from Oxidative Damage via Relieving Mitochondrial Dysfunction and Endoplasmic Reticulum Stress. J. Anim. Sci. Biotechnol..

[34] Samo S.P., Malhi M., Kachiwal A.B., Gadahi J.A., Parveen F., Kalhoro N.H., Lei Y. (2020). Supranutritional Selenium Level Minimizes High Concentrate Diet-Induced Epithelial Injury by Alleviating Oxidative Stress and Apoptosis in Colon of Goat. BMC Vet. Res..

[35] Bano I., Hassan M.F., Kieliszek M. (2025). A Comprehensive Review of Selenium as a Key Regulator in Thyroid Health. Biol. Trace Elem. Res..

[36] Wang F., Zha Z., He Y., Li J., Zhong Z., Xiao Q., Tan Z. (2023). Genome-Wide Re-Sequencing Data Reveals the Population Structure and Selection Signatures of Tunchang Pigs in China. Animals.

[37] Wang Z., Zhong Z., Xie X., Wang F., Pan D., Wang Q., Pan Y., Xiao Q., Tan Z. (2024). Detection of Runs of Homozygosity and Identification of Candidate Genes in the Whole Genome of Tunchang Pigs. Animals.

[38] Wang X., Zhang Y., Wen Q., Wang Y., Wang Z., Tan Z., Wu K. (2020). Sex Differences in Intestinal Microbial Composition and Function of Hainan Special Wild Boar. Animals.

[39] Li X., Xie F., Li R., Li L., Ren M., Jin M., Zhou J., Wang C., Li S. (2024). Integrated 4D Analysis of Intramuscular Fat Deposition: Quantitative Proteomic and Transcriptomic Studies in Wannanhua Pig Longissimus Dorsi Muscle. Animals.

[40] Oyelami F.O., Zhao Q., Xu Z., Zhang Z., Sun H., Zhang Z., Ma P., Wang Q., Pan Y. (2020). Haplotype Block Analysis Reveals Candidate Genes and QTLs for Meat Quality and Disease Resistance in Chinese Jiangquhai Pig Breed. Front. Genet..

[41] Liu X., Lyu W., Liu L., Lv K., Zheng F., Wang Y., Chen J., Dai B., Yang H., Xiao Y. (2021). Comparison of Digestive Enzyme Activities and Expression of Small Intestinal Transporter Genes in Jinhua and Landrace Pigs. Front. Physiol..

[42] (2020). Nutrient Requirements of Swine.

[43] (2016). National Food Safety Standard—Determination of Moisture in Foods.

[44] (2025). National Food Safety Standard—Determination of Protein in Food.

[45] (2025). National Food Safety Standard—Determination of Fat in Foods.

[46] (2016). National Food Safety Standard—Determination of Ash in Foods.

[47] Huang L., Guo Q., Wu Y., Jiang Y., Bai H., Wang Z., Chen G., Chang G. (2023). Carcass Traits, Proximate Composition, Amino Acid and Fatty Acid Profiles, and Mineral Contents of Meat from Cherry Valley, Chinese Crested, and Crossbred Ducks. Anim. Biotechnol..

[48] Li Z., Lv S., Liu Y., Cao M., Zhang H., Hao Q. (2026). Transcriptome and Metabolome Analyses Reveal the Accumulation Mechanism of Carbohydrates during Paeonia Ostii Seed Development. Biomolecules.

[49] Li L., Zhao Y., Guo Y., Shi B., Zhang J., Meng F., Hui F., Zhang Q., Guo X., Yan S. (2026). Integration of Serum and Liver Metabolomics with Antioxidant Biomarkers Elucidates Dietary Energy Modulation of the Fatty Acid Profile in Donkey Meat. Antioxidants.

[50] Shen L., Du J., Xia Y., Tan Z., Fu Y., Yang Q., Li X., Tang G., Jiang Y., Wang J. (2016). Genome-Wide Landscape of DNA Methylomes and Their Relationship with mRNA and miRNA Transcriptomes in Oxidative and Glycolytic Skeletal Muscles. Sci. Rep..

[51] Moreira C., Bonagurio L., Esteves L., Sitanaka N., Pozza P. (2021). Dietary Net Energy Mainly Affects Growth Performance and Pork Quality of Finishing Pigs. Sci. Agric..

[52] Yang Y., Liu H., Zou D., Ji F., Lv R., Wu H., Zhou H., Ren A., Xu T., Hou G. (2024). Polystyrene Microplastics Exposure Reduces Meat Quality and Disturbs Skeletal Muscle Angiogenesis via Thrombospondin 1. Food Res. Int..

[53] Chang C., Zhang Q.Q., Wang H.H., Chu Q., Zhang J., Yan Z.X., Liu H.G., Geng A.L. (2023). Dietary Metabolizable Energy and Crude Protein Levels Affect Pectoral Muscle Composition and Gut Microbiota in Native Growing Chickens. Poult. Sci..

[54] Li Y., Ma Q., Li M., Liu W., Liu Y., Wang M., Wang C., Khan M.Z. (2025). Non-Bovine Milk as Functional Foods with Focus on Their Antioxidant and Anti-Inflammatory Bioactivities. Antioxidants.

[55] Zhang J., Yin J., Zhou X., Li F., Ni J., Dong B. (2008). Effects of Lower Dietary Lysine and Energy Content on Carcass Characteristics and Meat Quality in Growing-Finishing Pigs. Asian-Australas. J. Anim. Sci..

[56] Talarico M.C.R., Derchain S., da Silva L.F., Sforça M.L., Rocco S.A., Cardoso M.R., Sarian L.O. (2024). Metabolomic Profiling of Breast Cancer Patients Undergoing Neoadjuvant Chemotherapy for Predicting Disease-Free and Overall Survival. Int. J. Mol. Sci..

[57] Wu C., Wu H., Jin R., Liang J., Tong X. (2026). Characterization of the Flavor Profile and Microbial-Driven Mechanism of Characteristic Flavor Formation in Yuxi Taihe Douchi. Sci. Rep..

[58] Yang Z., Hasan M.S., Humphrey R.M., Htoo J.K., Liao S.F. (2021). Changes in Growth Performance, Plasma Metabolite Concentrations, and Myogenic Gene Expression in Growing Pigs Fed a Methionine-Restricted Diet. Front. Biosci..

[59] Yang B., Shen P., Xu Z., Yang J., Song B., Jiang H., Chai J., Zhao J., Deng F., Li Y. (2025). Functional and Compositional Changes in Ileal Microbiota in Piglets during the Nursing Period Revealed by 16s rRNA Gene and Metagenomics. Animals.

[60] Yao D., Bao L., Wang S., Tan M., Xu Y., Wu T., Zhang Z., Gong K. (2024). Isoliquiritigenin Alleviates Myocardial Ischemia-Reperfusion Injury by Regulating the Nrf2/HO-1/SLC7a11/GPX4 Axis in Mice. Free Radic. Biol. Med..

[61] Wang J., Wang N., Qi M., Li J., Tan B. (2022). Glutamine, Glutamate, and Aspartate Differently Modulate Energy Homeostasis of Small Intestine under Normal or Low Energy Status in Piglets. Anim. Nutr..

[62] Zhi S.-M., Fang G.-X., Xie X.-M., Liu L.-H., Yan J., Liu D.-B., Yu H.-Y. (2020). Melatonin Reduces OGD/R-Induced Neuron Injury by Regulating Redox/Inflammation/Apoptosis Signaling. Eur. Rev. Med. Pharmacol. Sci..

[63] Liu Y., Yin S., He Y., Tang J., Pu J., Jia G., Liu G., Tian G., Chen X., Cai J. (2023). Hydroxy-Selenomethionine Mitigated Chronic Heat Stress-Induced Porcine Splenic Damage via Activation of Nrf2/Keap1 Signal and Suppression of NFκb and STAT Signal. Int. J. Mol. Sci..

[64] Song Y., Qu Y., Mao C., Zhang R., Jiang D., Sun X. (2023). Post-Translational Modifications of Keap1: The State of the Art. Front. Cell Dev. Biol..

[65] Li E., Li C., Horn N., Ajuwon K.M. (2023). Quercetin Attenuates Deoxynivalenol-Induced Intestinal Barrier Dysfunction by Activation of Nrf2 Signaling Pathway in IPEC-J2 Cells and Weaned Piglets. Curr. Res. Toxicol..

[66] Ayuso M., Irwin R., Walsh C., Van Cruchten S., Van Ginneken C. (2021). Low Birth Weight Female Piglets Show Altered Intestinal Development, Gene Expression, and Epigenetic Changes at Key Developmental Loci. FASEB J..

[67] Zhang Z., Dong S., Li J., Aizezi M., Huang P., Abula S., Mai Z., Liu D., Wusiman A. (2024). Effects of Lagenaria Siceraria (Molina) Standl Polysaccharides on Growth Performance, Immune Function, Cecum Microorganisms and Short-Chain Fatty Acids in Broilers. Front. Vet. Sci..

[68] Jang K.B., Kim S.W. (2022). Role of Milk Carbohydrates in Intestinal Health of Nursery Pigs: A Review. J. Anim. Sci. Biotechnol..

[69] Bekebrede A.F., de Boer V.C.J., Gerrits W.J.J., Keijer J. (2023). Functional and Molecular Profiling of Fasted Piglets Reveals Decreased Energy Metabolic Function and Cell Proliferation in the Small Intestine. Am. J. Physiol. Gastrointest. Liver Physiol..

[70] Adebowale T., Shunshun J., Yao K. (2019). The Effect of Dietary High Energy Density and Carbohydrate Energy Ratio on Digestive Enzymes Activity, Nutrient Digestibility, Amino Acid Utilization and Intestinal Morphology of Weaned Piglets. J. Anim. Physiol. Anim. Nutr..

[71] Li Y., Fu X., Sun F., Dong M., Wang Y., Wang Y., Liu Q. (2026). Metabolomic and Metagenomic Insights into WFBG-Mediated Regulation of Gut Microbiota and Metabolism in Broilers. Appl. Environ. Microbiol..

